# Supplementary material for: The Spectrum of Still’s Disease: A Comparative Analysis of Phenotypic Forms in a Cohort of 238 Patients
Source: J Clin Med. 2022 Nov 12;11(22):6703. doi: 10.3390/jcm11226703 (PMC9697610; doi:10.3390/jcm11226703)
Supplement: Supplementary file 1 [file jcm-11-06703-s001.zip › jcm-1991445-supplementary.pdf]

Table S1. Prognostic factors associated with favorable outcomes

		Favorable* (n=172)	Unfavorable § (n=66)	p-values (univariate)
**Epidemiology**				
	Men/Women	77/95	25/41	0.415
	Caucasian origin, N (%)	96/137 (70.1%)	36/49 (73.5%)	0.790
	sJIA/AOSD	64/108	22/44	0.684
**Evolution**				
	Chronic/Systemic	32/133	23/26	**<0.001**
**Clinical features**				
	Fever, N (%)	166/170 (97.6%)	56/65 (86.2%)	**0.002**
	Joint involvement N (%)	153/170 (90.0%)	57/66 (86.4%)	0.569
	Skin rash, N (%)	132/169 (78.1%)	35/64 (54.7%)	**0.001**
	Sore throat, N (%)	87/163 (53.4%)	30/62 (48.4%)	0.603
	Myalgia, N (%)	54/163 (33.1%)	18/62 (29.0%)	0.570
	Lymphadenopathy, N (%)	60/134 (44.8%)	21/45 (46.7%)	0.962
	Splenomegaly, N (%)	22/141 (15.6%)	7/51 (13.7%)	0.926
	Hepatomegaly, N (%)	22/138 (15.9%)	5/50 (10.0%)	0.429
	Pericarditis, N (%)	29/169 (17.2%)	12/64 (18.8%)	0.927
	Pleurisy, N (%)	24/172 (14.0%)	8/66 (12.1%)	0.874
	Digestive involvement, N (%)	30/166 (18.1%)	12/63 (19.0%)	1.000
**Biological characteristics**	WBCs, median [IQR], G/L	16.0 [12.1;20.4]	15.0 [10.5;20.0]	0.216
	PMNs, median [IQR], G/L	12.6 [9.47;16.1]	11.9 [7.03;16.4]	0.455
	Platelets, median (IQR), G/L	374 [267;475]	388 [264;550]	0.488
	Serum ferritin, median [IQR], μg/L	2187 [529;8400]	2400 [788;6000]	0.822
	FGF, median [IQR], %	14.0 [9.75;22.2]	17.0 [13.0;25.0]	0.338
	CRP, median [IQR], mg/L	165 [98.5;244]	164 [91.8;248]	0.787
	AST, median [IQR], U/L	40.0 [26.5;76.0]	36.5 [24.2;87.2]	0.518
	ALT, median [IQR], U/L	26.0 [14.0;75.5]	39.0 [15.0;77.0]	0.469
	LDH, median [IQR], U/L	373 [247;493]	513 [280;720]	**0.039**
	ANA, N (%)	31/139 (22.3%)	11/52 (21.2%)	1.000

* Favorable: patients who had achieved recovery or controlled disease.

§ Unfavorable: patients who did not fulfill favorable outcome criteria.

ANA: antinuclear autoantibody; ALT: alanine transaminase; AOSD: adult-onset Still's disease; AST: aspartate transaminase; CRP: C-reactive protein; FGF: Ferritin glycosylated fraction; LDH: lactate dehydrogenase; PMNs: polymorphonuclear neutrophils; sJIA: systemic juvenile idiopathic arthritis; WBCs: white blood cells.

Table S2. Prognostic factors associated with systemic vs. chronic course in adjusted analysis*

Prognostic factors	Odds ratio [95% CI]	p-values
Sex	1.89 [0.94-3.93]	0.08
Sore throat	0.36 [0.11-1.12]	0.08
Joint involvement	0.35 [0.09-1.13]	0.10
Lymphadenopathy	1.23 [0.57-2.67]	0.60
Splenomegaly	**3.86 [1.14-18.07]**	**0.05**
Digestive involvement	2.36 [0.92-6.88]	0.09
PMNs	**1.09 [1.03-1.16]**	**0.007**
Serum ferritin	1.00 [1.00-1.00]	0.21
MAS	**7.81 [1.43-146.54]**	**0.05**
Antibiotic therapy prior to diagnosis	1.37 [0.69-2.70]	0.36

*variables tested in the adjusted analysis: sex, MAS, skin rash, sore throat, lymphadenopathy, splenomegaly, digestive involvement, joint involvement, PMN count, serum ferritin level, antibiotic therapy prior to diagnosis.
MAS: macrophage activation syndrome; PMNs: polymorphonuclear neutrophils.

Table S3. Distribution of epidemiological, clinical and laboratory features according to the disease phenotype in sJIA and AOSD patients

Characteristics		sJIA (n=86)		AOSD (n=152)	
		Chronic (n=22)	Systemic (n=56)	Chronic (n=33)	Systemic (n=103)
**Epidemiology**					
	Men/Women	7/15	27/29	12/21	47/56
	Median age at diagnosis, y [IQR]	8.2 [1.6-13.5]	6.4 [3.3-12.3]	43.0 [29.9-57.4]	36.0 [26.8-45.8]
	Median diagnostic delay, d [IQR]	52 [42-77]	28 [20-64]	45 [17-240]	31 [17-74]
	Caucasian origin, N (%)	7/15 (46.7%)	33/45 (73.3%)	22/26 (84.6%)	54/83 (65.1%)
**Classification criteria**					
	ILAR, N (%)	7/15 (46.7%)	28/51 (54.9%)	-	-
	PRINTO, N (%)	7/13 (53.8%)	33/51 (64.7%)	-	-
	Yamaguchi, N (%)	-	-	16/28 (57.1%)	65/99 (65.7%)
	Fautrel, N (%)	-	-	15/28 (53.6%)	73/99 (73.7%)
**Clinical features**					
	Fever, N (%)	19/22 (86.3%)	54/56 (96.4%)	31/33 (93.9%)	96/100 (96.0%)
	Fever > 39°C, N (%)	12/14 (85.7%)	39/44 (88.6%)	17/23 (73.9%)	83/92 (90.2%)
	Joint involvement N (%)	21/22 (95.4%)	48/56 (85.7%)	30/33 (90.1%)	90/101 (89.1%)
	Skin rash, N (%)	11/20 (55.0%)	46/56 (82.1%)	18/33 (54.5%)	77/100 (77.0%)
	Sore throat, N (%)	5/20 (25.0%)	13/54 (24.1%)	18/32 (56.3%)	71/96 (74.0%)
	Myalgia, N (%)	2/18 (11.1%)	14/54 (25.9%)	10/31 (32.3%)	40/98 (40.8%)
	Lymphadenopathy, N (%)	4/12 (33.3%)	22/43 (51.2%)	7/23 (30.4%)	37/82 (45.1%)
	Splenomegaly, N (%)	1/15 (6.67%)	9/46 (19.7%)	0/28 (0.0%)	15/83 (18.1%)
	Hepatomegaly, N (%)	2/15 (13.3%)	9/46 (19.7%)	1/28 (3.6%)	10/80 (12.5%)
	Pericarditis, N (%)	2/20 (10.0%)	13/56 (23.2%)	6/32 (18.6%)	17/102 (16.7%)
	Pleurisy, N (%)	0/22 (0.0%)	11/56 (19.6%)	5/33 (15.2%)	15/103 (14.6%)
	Digestive involvement, N (%)	2/20 (10.0%)	12/56 (21.4%)	2/32 (6.3%)	22/98 (22.4%)
	Weight loss, N (%)	2/12 (16.7%)	7/29 (24.1%)	9/20 (45.0%)	23/78 (29.4%)
**Biological characteristics**					
	WBCs, median [IQR], G/L	15.4 [8.8-17.0]	18.9 [15.7-23.3]	13.6 [9.2-17.1]	15.4 [11.5-18.8]
	PMNs, median [IQR], G/L	7.0 [3.8-12.1]	15.0 [11.7-19.1]	10.9 [7.1-14.0]	12.6 [9.0-15.4]
	Hemoglobin, mean (SD), g/L	104 (±12.8)	104 (±13.5)	124 (±17.0)	119 (±17.4)
	Platelets, median [IQR], G/L	390 [319-421]	448 [354-574]	342 [286-541]	314 [231-458]
	Serum ferritin, median [IQR],	463 [107-5479]	1302 [455-3323]	2014 [513-5000]	3380 [1104-12500]
	FGF, median [IQR], %	-	-	21 [14-29]	14 [9-22]
	FGF <20%, N (%)	2/3 (66.7%)	1/2 (50.0%)	11/23 (47.8%)	59/82 (72.0%)
	CRP, median [IQR], mg/L	91 [30-156]	136 [86-197]	174 [93-233]	194 [112-281]
	AST, median [IQR], U/L	36 [26-41]	33 [24-46]	27 [21-58]	56 [30-106]
	ALT, median [IQR], U/L	21 [16-33]	12 [8-19]	31 [16-52]	59 [24-124]
	GGT, median [IQR], U/L	24 [14-40]	18 [14-29]	59 [24-120]	109 [48-219]
	LDH, median [IQR], U/L	287 [237-340]	408 [299-498]	298 [206-420]	379 [245-575]
	PT, median [IQR], %	90 [80-96]	65 [56-77]	80 [75-86]	79 [69-91]
	Fibrinogen, mean (SD), g/L	5.0 (±2.0)	6.0 (±2.0)	6.9 (±2.3)	6.6 (±2.3)
	Triglycerides, median [IQR],	1.76 [1.15-1.90]	1.39 [1.03-2.13]	1.51 [1.10-1.84]	1.57 [1.24-2.20]
	Rheumatoid factor, N (%)	0/11 (0.0%)	2/20 (10.0%)	3/22 (13.6%)	5/73 (6.8%)
	ANA, N (%)	9/14 (64.3%)	4/39 (10.3%)	6/30 (20.0%)	20/90 (22.2%)

ANA: antinuclear autoantibody; ALT: alanine transaminase; AOSD: adult-onset Still's disease; AST: aspartate transaminase; CRP: C-reactive protein; d: days; FGF: Ferritin glycosylated fraction ; GGT: gamma-glutamyltransferase; ILAR: International League of Associations for Rheumatology; LDH: lactate dehydrogenase; PMNs: polymorphonuclear neutrophils; PRINTO: Pediatric Rheumatology

International Trials Organization; PT: prothrombin time; sJIA: systemic juvenile idiopathic arthritis; WBCs: white blood cells; y: years.
